# Organic UV Filters Induce Toll-like-Receptors and Related Signaling Pathways in Peripheral Blood Mononuclear Cells of Juvenile Loggerhead Sea Turtles (*Caretta caretta*)

**DOI:** 10.3390/ani12050594

**Published:** 2022-02-27

**Authors:** Paolo Cocci, Gilberto Mosconi, Francesco Alessandro Palermo

**Affiliations:** School of Biosciences and Veterinary Medicine, University of Camerino, Via Gentile III Da Varano, I-62032 Camerino, MC, Italy; paolo.cocci@unicam.it (P.C.); gilberto.mosconi@unicam.it (G.M.)

**Keywords:** UV filters, loggerhead sea turtles, endocrine disruptors, toll-like receptors, pro-inflammatory cytokines, immunotoxicity

## Abstract

**Simple Summary:**

Emerging environmental contaminants, such as sunscreen agents, have been broadly identified in marine ecosystems. Thus, the present work aims to investigate whether organic UV filters cause immunotoxic effects in juvenile loggerhead sea turtles (*Caretta caretta*). We found that loggerhead sea turtles showing high circulating levels of organic UV filters manifested increased expression of genes involved in inflammatory responses, probably due to contaminant-induced oxidative damage.

**Abstract:**

Recent evidence suggests that exposure to organic ultraviolet filters (UV filters) is associated with dysregulated neuroendocrine-immune homeostasis. Marine species are likely to be among the most vulnerable to UV filters due to widespread diffusion of these chemicals in the aquatic environment. In the present study, the effects of UV filter bioaccumulation on toll-like-receptors (TLRs) and related signaling pathways were investigated in peripheral blood mononuclear cells (PBMCs) of juvenile loggerhead sea turtles (*Caretta caretta*). We found that the expression of both TLR1 and TLR2 was significantly increased in UV-filter exposed turtles compared to control animals. Similarly, the signaling pathway downstream of activated TLRs (i.e., Ras-related C3 botulinum toxin substrate 1 (RAC1), Phosphoinositide 3-kinase (PI3K), serine/threonine-protein kinase (AKT3), and nuclear factor κB (NF-κB)) was significantly up-regulated, leading to an enhanced transcription of pro-inflammatory cytokines. In addition, we demonstrated that high levels of plasma UV filters increased lipid peroxidation in sea turtles’ PBMCs. Our results indicated that UV filters affected the inflammatory responses of PBMCs via modulation of the TLR/NF-κB signaling pathway and provided a new insight into the link between exposure to sunscreen agents and sea turtle health.

## 1. Introduction

Organic ultraviolet filters (UV filters) are compounds containing single or multiple aromatic structures attached to hydrophobic groups which are widely used in sunscreens and cosmetic products [1]. Previous studies, mainly conducted in Europe, demonstrated that 98% of sunscreen products contain from three to eight organic UV filters and 71% of personal care products contain at least one [2,3]. Recently, UV filters have been detected in 86% of sunscreens and in 53% of other cosmetic products in the Asian market [4]. Because of their increasing industrial production, the aquatic environment has been overloaded with these chemicals that can be widely detected in water, sediments, and biota [5]. The presence and potential negative effects of organic UV filters in the marine ecosystem have previously been reported in several studies [6,7,8,9]. Recent data also indicate that UV filters have light lipophilicity and can be considered pseudopersistent environmental contaminants, which, however, have the ability to bioaccumulate in various aquatic organisms [10]. The risk posed by these compounds to marine organisms stems from, among other things, their potential genotoxicity, mutagenicity, and endocrine disruption at environmentally relevant concentrations. In this regard, current attention has mainly been given to the reproductive and developmental toxicity of benzophenone (BP)-type UV filters in fish [11,12,13]. In contrast, studies on the potential of these chemicals to alter immune responses in aquatic organisms are still scarce. Studying the immune responses of wild animals in their environment has acquired an increased importance since many infectious diseases may have been worsened by pollutant-mediated alteration of the endocrine-immune interactions [14]. In fact, there is evidence that environmental contamination is associated with increased incidence of immunosuppression in several vertebrate models [15], including reptiles [16]. It is possible that environmental pollutant-induced functional modulation of the endocrine-immune interactions may worsen the effects of infectious diseases, thereby leading to the development of serious illnesses. Accumulating lines of evidence suggest a correlation between oxidative stress from exposure to environmental pollutants and immune functions which support the role of reactive oxygen species (ROS) in the induction of immunotoxicity [17,18,19]. In this regard, we have previously investigated the relationship between gene biomarker expression and organic UV filter accumulation in juvenile loggerheads and found that total sunscreen agent concentration was strongly positively associated with gene biomarkers of inflammation and oxidative stress [8]. There is evidence that under conditions of oxidative stress, endogenous molecules that are key mediators of the innate immune system (i.e., damage-associated molecular patterns, DAMPs) are released from stressed cells. The release of DAMP molecules is likely to activate signaling pathways that are involved in mediating the resulting inflammatory response. These signaling pathways involve, among other things, the activation of toll-like receptors (TLRs) [20]. The TLR family of receptors constitute one of the first lines of the immune defense system leading to the activation of the acquired immune response [21]. Although the characteristics of TLRs have been widely investigated in mammals, there is limited information regarding the functional role of these receptors in reptiles, especially in sea turtles. Evolutionary studies have shown that TLRs are generally conserved, and a series of TLR family genes has been found in many species [22]. Zhou et al. [23] and Hu et al. [24] have provided a functional and expression analysis of different TLRs in the Chinese soft-shelled turtle *Pelodiscus sinensis*. In this regard, the phylogenetic analyses have demonstrated high similarities in TLR homologs among *P. sinensis* and other turtle species such as *C. mydas, C. picta,* and *T. mexicana* [25]. Therefore, to better understand the relationship between organic UV filter exposure and immunotoxicity, we examined the expression patterns of genes related to the TLR signaling pathway (KEGG pathway: cmy 04620), because these receptors play a key role in mediating the inflammatory response to conditions of oxidative stress. Specifically, we evaluated toll-like receptor–mediated nuclear factor kappa B (NF-κB) activation through the signaling cascade composed of Ras-related C3 botulinum toxin substrate 1 (RAC1), Phosphoinositide 3-kinase (PI3K), and RAC serine/threonine-protein kinase (AKT3) in peripheral blood mononuclear cells (PBMCs) of juvenile loggerhead sea turtles (*Caretta caretta*) showing high circulating levels of organic UV-filters. PBMCs were selected as these cells can be easily and safely collected from sea turtles and can serve as models for monitoring the transcriptomic profile indicative of immunotoxic effects. NF-κB transcriptional activity was further investigated by evaluating the expression of various pro-inflammatory genes such as tumor necrosis factor alpha (TNF-α), IL-6, and IL-12.

## 2. Materials and Methods

### 2.1. Samples Handlings

**Figure 1:**
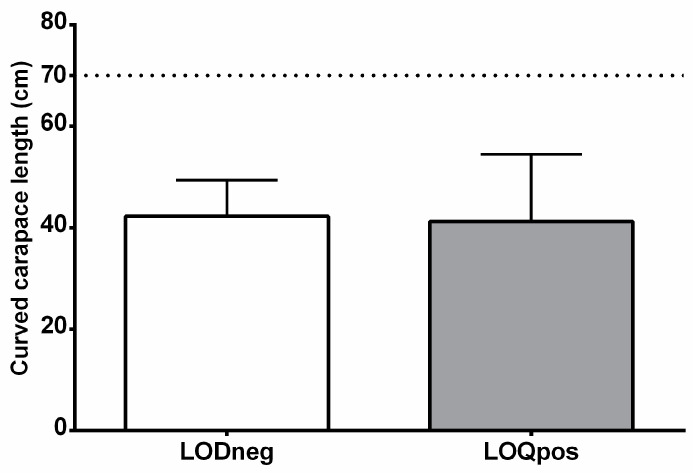
Size distribution of the two groups of loggerhead turtles analyzed in this study. Values are given as mean + SD. Dot line shows CCL threshold for identifying immature specimens [26,27,30].

Thirty-two juvenile loggerhead sea turtles (*C. caretta*) recovered along the Italian coasts (North and Central Adriatic Sea) were enrolled for this study [8]. The healthy condition of sea turtles was determined on the basis of hematological values and individual clinical examination by veterinary rehabilitation experts of the regional center of Care and Rehabilitation for Sea Turtles (Fondazione Cetacea onlus) Riccione, Italy. Sea turtles with traumatic injuries, eye/skin diseases, and infections were not enrolled in the study. Curved carapace lengths (CCLs) of the selected sea turtles were measured in order to confirm the juvenile status (Figure 1) [26,27,28]. Animals were divided into two subgroups according to the plasma levels of UV filters found in our previous study [4]. We selected the following two categories: (1) LODneg if the plasma level of each UV filter was below LODs (<0.15 μg mL^−1^ (Ensulizole); <0.30 μg mL^−1^ (Ethyl salicylate); <0.20 μg mL^−1^ (Benzophenone-3); <0.40 μg mL^−1^ (Homosalate)) and (2) LOQpos if the concentration of UV filters was above LOQs (>0.40 μg mL^−1^ (Ensulizole); >0.80 μg mL^−1^ (Ethyl salicylate); >0.60 μg mL^−1^ (Benzophenone-3); >1.00 μg mL^−1^ (Homosalate). The LODneg group involved 13 specimens while the LOQpos group contained 19 specimens showing an average ΣUV filter concentration of 10.23 ± 9.92 μg mL^−1^; min–max: 1.03–31.75 μg mL^−1^). Briefly, blood was taken from the dorsal cervical sinus and processed according to the procedure described by Cocci et al. [8]. Peripheral blood mononuclear cells were immediately collected and kept at −80 °C until processed for molecular studies. Animal manipulation was carried out using standard operating actions as previously described [29] and according to the D.G.R. 563/08–D.G.R. 664/08.

### 2.2. Real-Time Reverse Transcription PCR (qRT-PCR)

Total RNA was extracted from nucleated blood cells using the Trifast™ kit (EuroClone) according to the manufacturer’s specifications. The complementary DNA (cDNA) was synthesized from 3 μg of total RNA using the All-In-One 5× RT MasterMix kit including genomic DNA (gDNA) removal (abm^®^). The reverse transcription reaction was set-up by adding 4 µL 5X All-In-One RT MasterMix and Nuclease-free H_2_O. Samples were incubated at 37 °C for 15 min and then at 60 °C for 30 min. The reaction was inactivated at 85 °C for 3 min. A SYBR Green Real-Time PCR assay (ABI 7300) was performed for the molecular analyses with primers for IL-6, IL-12, RAC1, AKT3, TNF-α, PI3K, TLR1, TLR2, NF-kB, and NF-kappa-B inhibitor alpha (IkBα) target genes designed using the Primer-BLAST tool (https://www.ncbi.nlm.nih.gov/tools/primer-blast/) according to the *Chelonia mydas* gene specific sequences (Table 1). Ribosomal 18 s RNA was selected as a reference gene [31]. The reaction mixture contained 10 μL BlasTaq™ 2X qPCR MasterMix (abm^®^), 0.5 µL of primers (both 10 µM), 2 µL cDNA template and Nuclease-free H_2_O. Thermal cycling for IL-6, IL-12, RAC1, AKT3, TNF-α, PI3K, TLR1, TLR2, and 18s reactions consisted of 3 min at 95 °C, followed by 40 cycles of 15 s at 95 °C and 60 s at 60 °C. Thermo-cycling for NF-kB and IkBα reactions consisted of 3 min at 95 °C, followed by 40 cycles of 20 s at 95 °C and 60 s at 58 °C. The specificity of the primer pairs was confirmed by applying the melting curve analysis produced by the ABI 7300 software and verified with agarose gel electrophoresis (Table 1). All used primers showed high specificity and sensitivity, indicating that the cross-species primers can correctly amplify the target genes in *C. caretta* [8,31].

### 2.3. Lipid Peroxidation (LPO)

Lipid peroxidation was assessed following the procedure described by Cocci et al. [31]. Nucleated blood cells were homogenized in 0.9% NaCl and incubated for 15 min at 37 °C. A mixture of HCl/trichloroacetic acid and thiobarbituric acid were added to the sample and incubated at 100 °C for 10 min. Following centrifugation at 4000 rpm, the supernatant was collected and the absorbance (535 nm) was detected.

### 2.4. Statistical Analyses

Data analysis was performed using GraphPad Prism version 8 software (GraphPad Software, Inc., La Jolla, CA, USA). q-PCR results were expressed as normalized fold change corrected for 18s rRNA and with respect to the LODneg group. Data were first examined for their fit to a normal distribution and homogeneity of variance using Kolmogorov–Smirnov and Levene median tests. Data were then analyzed using the Student t test. The significance cut-off for the Student’s *t*-test was taken as *p* < 0.05.

## 3. Results

**Figure 2:**
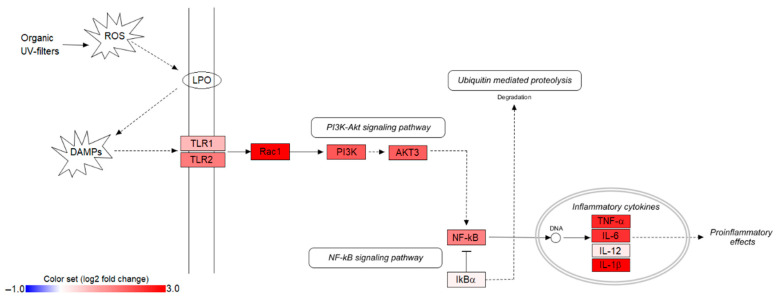
Illustration of the TLR/NF-κB signaling pathway, along with the log2 fold changes of 11 pathway genes in UV filter exposed loggerheads (LOQpos). Interleukin 1 beta (IL-1β) gene expression dataset was collected from our previous work, Cocci et al. [8]. ROS: reactive oxygen species; DAMPs: damage-associated molecular patterns; LPO: lipid peroxidation.

To assess the potential role of TLRs in response to organic UV filter accumulation, the expression levels of selected genes in the TLR-mediated signaling pathways were investigated. We found an increase of transcription for most of the genes tested, except IκBα and IL-12 (Figure 2).

**Figure 3:**
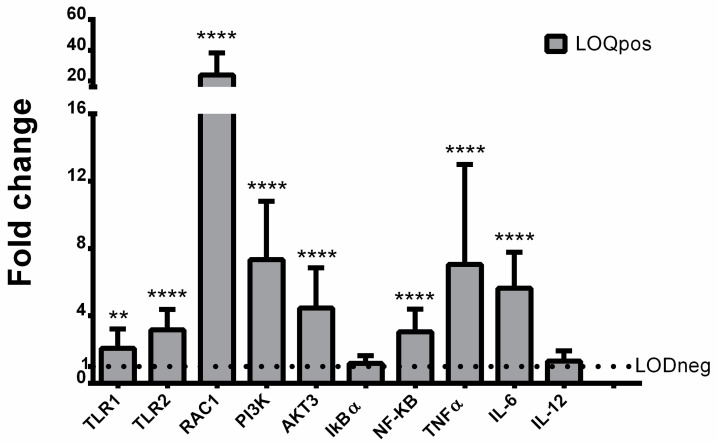
Mean mRNA fold change (+SD) of toll-like-receptors (TLRs) 1 and 2, Ras-related C3 botulinum toxin substrate 1 (RAC1), Phosphoinositide 3-kinase (PI3K), serine/threonine-protein kinase (AKT3), nuclear factor κB (NF-κB), inhibitory protein kappa B alpha (IkBα), tumor necrosis factor alpha (TNF-α), interleukin 6 (IL-6), and IL-12 relative to LODneg group (adjusted average LODneg value is 1) is shown. **, *p* ≤ 0.01; **** *p* ≤ 0.0001 (unpaired *t*-tests).

The expression of both TLR1 and TLR2 was significantly increased in UV- filter exposed turtles with respect to control animals (Figure 3).

**Figure 4:**
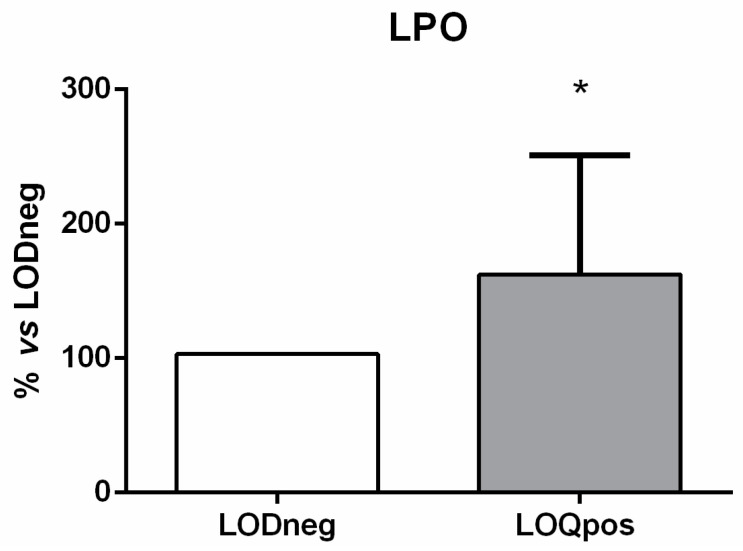
Effects of UV filters on lipid peroxidation (LPO) in loggerhead peripheral blood mononuclear cells. Results are expressed as percentage (%) of mean + SD versus LODneg group. * *p* ≤ 0.05 (unpaired *t*-tests).

Similarly, the signaling pathway downstream of activated TLRs (i.e., RAC1, PI3K, Akt, and NF-κB) was significantly up-regulated (Figure 3). Of these four genes, RAC1 was the most highly expressed, showing a 24-fold increase. On the contrary, the mRNA expression of IκBα did not exhibit any significant variation compared with the LODneg group (Figure 3). The gene transcription of NF-κB targets was also significantly induced in samples showing high content of UV filters. Indeed, the mRNA levels of TNF-α and IL-6 were found to be increased from 5-fold up to 8-fold, whereas the expression of IL-12 was not modulated compared with the LODneg group (Figure 3). In addition, PBMCs were also examined for oxidative damage measured as LPO. Data indicated that LPO levels were significantly increased in turtles showing high content of UV filters compared to LODneg animals (Figure 4).

## 4. Discussion

TLRs are involved in mediating the inflammatory response activated by infectious agents, thus playing a pivotal role in innate immunity [32]. However, similarities between the signaling pathways triggered by microbial products and oxidative stress have been recently suggested [33,34]. There is evidence that bacterial cell wall components, activated DAMPs, and proinflammatory cytokines share a common TLR-mediated signaling pathway leading to NF-κB nuclear translocation [35]. Although certain consequences of TLR-dependent activation of transcription factors are known, the molecular mechanisms of intracellular signaling are largely undefined, especially in reptiles. Our results indicate that TLR signaling to NF-κB is activated in loggerheads with high plasma levels of UV filters. In addition, these animals show increased LPO levels compared to values found in the LODneg group. LPO is directly involved in tissue injuries and especially in the tissue damage caused by exposure to toxic substances. In this regard, previous studies in our lab proved that chronic high-level exposure to PAH mixtures triggered reactive oxygen species (ROS) production leading to LPO in the whole blood of juvenile loggerheads [31]. Recent studies have shown that the lipid peroxidation modification of proteins can induce the innate immune system working as DAMPs [36,37]. West et al. [38] observed that lipid peroxidation-derived adducts were recognized by TLR2/TLR1 and TLR2/TLR6 heterodimers, which are thus responsible for bridging inflammation, oxidative stress, and innate immunity. Our results suggested that UV filter accumulation caused ROS-induced lipid peroxidation in PBMCs of juvenile loggerheads. This phenomenon is most likely to be involved in potential release of DAMPS, which in turn activate TLR signaling pathways, thereby driving upregulation of downstream effective cytokines. The mRNA levels of TNF-α, IL-1β, and IL-6 were all upregulated in UV-filter exposed turtles, indicating activation of the NF-κB signaling module. In this last regard, our finding in the present work was consistent with the previous studies [39,40] and suggested that the PI3K/Akt pathway was involved in downstream NF-κB activation. Chen et al. [41] have demonstrated that maternal Disononyl Phthalate (DINP) exposure contributes to inducing airway inflammation in rat pups by upregulating the PI3K/Akt pathway. The same pathway was found to be involved in triggering the increase in TNF-α levels following treatment with Mono 2-ethylhexylphthalate MEHP [42]. Overall, these findings support the role of PI3K/Akt signaling in mediating environmental chemical (particularly phthalate)-induced toxicity. In this study, we also reported that the PI3K/Akt/ NF-κB pathway was potentially associated with the activation of RAC1. Our data clearly point to the UV filter-associated up-regulation of RAC1 as a crucial control point in the TLR signaling pathway. Several pieces of evidence suggest a role of RAC1 in regulating IL1-mediated NF-κB activation and expression of proinflammatory genes [43,44]. Jefferies and O’Neill [45] have shown the specific involvement of RAC1 in increasing the NF-kB transactivating potential of its p65 subunit without affecting the inhibitory subunit IkBα. Interestingly, we found that IκBα gene expression was not modified by exposure to UV-filters. IκBα acts as an inhibitor of NF-κB activity, blocking its nuclear localization and transcriptional activation [46]. It has been demonstrated that NF-κB activation requires initiating phosphorylation of IκBα [47]. Furthermore, this mechanism seems to be largely influenced by the parallel inhibition of phosphatase activity that can, among other things, also be attributed to ROS generation [48]. The difference in the transcription rate for IκBα compared to NF-κB allowed us to speculate about the potential role of UV filters in deregulating the transcriptional activation and degradation pathways of these molecules, thus repressing the NF-κB negative feedback. The induction of NF-κB plays a pivotal role in the inflammatory response, leading to transcription of gene coding for proinflammatory mediators. Consistently, the present study demonstrated that loggerheads exposed to UV filters showed increased expression of TNF-α, IL-1β, and IL-6. These results are in line with previous reports indicating that diverse environmental pollutants, particularly phthalates, possess the ability to exacerbate the inflammatory responses of macrophages by enhancing the levels of TNF-α, IL-1β, IL-6, and IL-8 [42,49,50]. Furthermore, the involvement of IκB/NF-κB signaling in inducing the transcript levels of inflammatory cytokines was demonstrated in PC12 cells exposed to tetrachlorobenzoquinone [51]. Our data are consistent with those by Ao et al. [52], who highlighted the ability of four organic UV filters to activate the NF-κB pathway, raising TNF-α and IL-6 levels. At present, studies of organic UV filter toxicity in aquatic organisms have focused mainly on the endocrine-disrupting potential [53]. Less is known about their immunotoxic characteristics. Our results are the first to demonstrate the involvement of the TLR/NF-κB pathway in mediating activation of the loggerhead sea turtle’s immune system in response to disturbances induced by UV filters. Disorders of the immune system may cause chronic susceptibility to infection. Indeed, several studies have demonstrated a correlation between exposure to endocrine-disrupting chemicals (EDCs) and the development of infectious diseases. For example, phthalates were found to induce cytokine production and immunoglobulin secretion [42], and to be related to the development of asthmatic inflammation [54].

## 5. Conclusions

Taken together, our data show that UV filter accumulation can activate the TLR/NF-κB pathway in loggerhead PBMCs, leading to the over-expression of TNF-α, IL-1β, and IL-6 genes. Thus, organic UV filters might exert, as other immunotoxic pollutants, a pro-inflammatory function which is likely to be triggered by ROS generation and oxidative stress.

## Figures and Tables

**Table 1:** Details of primers used in this study.

Gene	Primer Sequence (5′-3′)	GenBank	PCR Product (bp)	Melting Curve (°C)	Efficiencies (%)
IL-6	CAGTGATCATGCCAGACCCA TCGAACAGCCCTCACAGTTT	XM_043541083.1	143	83.0	98.3
IL-12	GGAACACCAGCCCATTGAGA CCACATGCTCACACTCAGGT	XM_007057267.4	122	85.5	93.4
RAC1	TTACACAGCGAGGCCTCAAG CCTTGTTCCGAGCAAAGCAC	XM_027823047.3	156	85.4	95.3
AKT3	AGTGACGTCGGGAGTTTTCC GCTACATGGAGCGAGCGTC	XM_037895339.2	174	87.7	94.8
TNF-α	TGAGCACCGAAAGTCTGGTC TCTGAAATGCAGCAGAGCGA	XM_027821468.3	155	90.3	96.7
PI3K	AGCGAGAGCTGAGGATCTTCTTT CATGCCAAACCTTCATTGCTTCC	XM_037909268.2	159	84.1	98.5
TLR1	TTAACTGAGCTGCCTGGGTG GGAATGGATTGTGCCCTCCT	XM_007059713.3	142	82.6	96.5
TLR2	TGGTGAAGAATGTGCCTGCT AGACCGTGCTTTACGTCTGG	XM_027821652.3	128	84.1	95.7
NF-kB	CGCGTGAGGCTCTTAAAATGG TGGTCCATCTGTTCGTAGTGG	XM_007054382.4	155	88.0	92.1
IkBα	CCAGGGGCCTTTAGGTAAGC GTTCCAACCTGCTGGCATTC	XM_037900614.2	112	80.8	96.1
18s rRNA	CGTTCTTAGTTGGTGGAGCG AACGCCACTTGTCCCTCTAA	HQ914786.1	124	85.3	100.9

## Data Availability

The datasets generated during and/or analysed during the current study are available from the corresponding author on request.
